# *Mauritia flexuosa* palm trees airborne mapping with deep convolutional neural network

**DOI:** 10.1038/s41598-021-98522-7

**Published:** 2021-10-04

**Authors:** Luciene Sales Dagher Arce, Lucas Prado Osco, Mauro dos Santos de Arruda, Danielle Elis Garcia Furuya, Ana Paula Marques Ramos, Camila Aoki, Arnildo Pott, Sarah Fatholahi, Jonathan Li, Fábio Fernando de Araújo, Wesley Nunes Gonçalves, José Marcato Junior

**Affiliations:** 1 Faculty of Engineering, Architecture, and Urbanism and Geography, Federal University of Mato Grosso do Sul (UFMS), Avenida Costa e Silva, Campo Grande, Mato Grosso do Sul 79070-900 Brazil; 2 Faculty of Engineering and Architecture and Urbanism, University of Western São Paulo (UNOESTE), Rodovia Raposo Tavares, km 572-Limoeiro, Presidente Prudente, São Paulo 19067-175 Brazil; 3 Post-Graduate Program in Environment and Regional Development, University of Western São Paulo (UNOESTE), Rodovia Raposo Tavares, km 572-Limoeiro, Presidente Prudente, São Paulo 19067-175 Brazil; 4 Department of Geography and Environmental Management, University of Waterloo (UW), Waterloo, ON N2L 3G1 Canada; 5 Post-Graduate Program in Agronomy, University of Western São Paulo (UNOESTE), Rodovia Raposo Tavares, km 572-Limoeiro, Presidente Prudente, São Paulo 19067-175 Brazil

**Keywords:** Computational biology and bioinformatics, Ecology

## Abstract

Accurately mapping individual tree species in densely forested environments is crucial to forest inventory. When considering only RGB images, this is a challenging task for many automatic photogrammetry processes. The main reason for that is the spectral similarity between species in RGB scenes, which can be a hindrance for most automatic methods. This paper presents a deep learning-based approach to detect an important multi-use species of palm trees (*Mauritia flexuosa*; i.e., Buriti) on aerial RGB imagery. In South-America, this palm tree is essential for many indigenous and local communities because of its characteristics. The species is also a valuable indicator of water resources, which comes as a benefit for mapping its location. The method is based on a Convolutional Neural Network (CNN) to identify and geolocate singular tree species in a high-complexity forest environment. The results returned a mean absolute error (MAE) of 0.75 trees and an F1-measure of 86.9%. These results are better than Faster R-CNN and RetinaNet methods considering equal experiment conditions. In conclusion, the method presented is efficient to deal with a high-density forest scenario and can accurately map the location of single species like the *M. flexuosa* palm tree and may be useful for future frameworks.

## Introduction

The unplanned development and land occupation in both urban and rural areas are the main reasons behind deforestation, contributing to environmental degradation in riparian zones and modifying the natural landscape. Multidisciplinary research is necessary to ascertain the population of vegetative species, identifying their locations and distribution patterns. Such information is essential for the management and conservation of vulnerable ecosystems, and mapping these environments may help governmental entities to control or mitigate environmental damage properly. In the last decade, remote sensing data have been widely applied for monitoring vegetation health^1^, biomass^2^, forest management^3^, biodiversity^4^, among others^5–8^. Thus, remote sensing approaches have been used to investigate areas with difficult terrain access, demonstrating great potential for the classification and detection of forest vegetation.

Remote sensing platforms can be embedded with different sensors such as RGB (Red–Green–Blue), multispectral and hyperspectral, LiDAR (Light Detection and Ranging), and others^9^. The identification of arboreous vegetation with remote sensing data depends on the spatial and spectral resolutions^10^. LiDAR sensors can produce accurate data on the height of the trees, which is an excellent variable to be adopted by automatic extraction methods in forest environments^11,12^. Multispectral and hyperspectral sensors have the advantage of recording the spectral divergence of the vegetation, which is important for enhancing differences between species configurations, health status, etc.^8,13^. Still, in recent years, high spatial resolution images acquired by RGB sensors have been used in many studies for vegetation identification^7,14–17^. These sensors have a relatively low cost in comparison with others but offer limited information regarding the spectral range.

For single-tree species mapping, the literature already investigated different methods by evaluating multispectral and hyperspectral data^18–20^, airborne LiDAR point clouds^21^, and multi-sensory data fusion^22,23^. Immitzer et al.^24^ were able to classify tree species in a temperate forest using satellite multispectral imagery. Franklin and Ahmed^25^ evaluated UAV (Unmanned Aerial Vehicle)-based multispectral image to map deciduous vegetation. Dalponte et al.^26^ used hyperspectral data to detect boreal tree species at pixel-level, achieving high accuracy for forest mapping. Most of these studies were conducted with hyperspectral sensors and LiDAR sensors. However, both hyperspectral and LiDAR data cost and process demand are non-attractive for rapid decision models. This is different from RGB sensors, which have a lower cost and are highly available. Guimarães et al.^27^ demonstrated that the majority of recent applications are implementing RGB imagery data in the vegetation detection scenario.

The visual inspection of remote sensing imagery is a time-consuming, labor-intensive, and biased task. Therefore, various studies have developed multiple methods regarding the automated extraction of the vegetation features^8,28,29^. Accurately mapping individual tree species in densely forested environments is still a challenging task, even for more robust methods. The increase in quality and quantity in remote sensing data, alongside the rapid improvement of technological resources, allowed for the development of intelligent methods in the computational vision community. By combining remote sensing data with artificial intelligence techniques, it is possible to properly map tree species and improve accuracy in applications regarding vegetation monitoring. In recent years, multiple frameworks have been implemented to assess the performance of such algorithms to accomplish this task^2,30,31^.

During the past years, the detection and extraction of trees in high-resolution imagery were performed with more traditional machine learning algorithms, like support vector machine (SVM), random forest (RF), artificial neural networks (ANN), and others^32–35^. They returned interesting outcomes in plenty of studies regarding vegetation analysis^8,36–40^. However, these learners (known as shallow learners) are limited due to data complexity and may return lower accuracy in comparison with deep learning methods. When considering adverse conditions in a given forest dataset, deeper methods are required.

Identifying individual species in a scene can be a challenging task. However, state-of-the-art deep learning-based methods should be capable of identifying single tree-species with an attractive accuracy and computational load even in RGB images. Recently, deep learning-based methods have been implemented in multiple remote sensing, specifically for image segmentation, classification, and object detection approaches^31,41,42^. Deep learning techniques are among the most recently adopted approaches to process remote sensing data^9,43^. In a general sense, deep learning can return better performance than shallow learners, especially in the presence of large quantities of data or if the input data is highly complex^44,45^.

In heavy-dense forested environments, the identification of single-tree species can become a challenge even for robust methods like current state-of-the-art deep networks. This motivated several studies to investigate the performance of deep learning methods to evaluate their performance on this task. A recently published research tested the performance of object detection using deep networks like YOLOv3^46^, RetinaNet^47^, and Faster-RCNN^48^ to detect tree canopy in RGB imagery covering an urban area^7^. Another study modified the VGG16^49^ to monitor the health conditions of vegetation^50^. A combination of LiDAR and RGB images was also used with the RetinaNet to identify tree-crowns in UAV images^16^. The DenseNet^51^ was also implemented multispectral data to classify tree species.

The spatial and spectral divergences between the tree and non-tree are essential for automatic methods^13^. In highly-dense scenarios like heavily forested areas, the individual detection of trees could be difficult. RGB sensors are not capable of providing the same amount of spectral data as multispectral or hyperspectral sensors, which offers a potential hindrance for automatic extraction methods. Nonetheless, state-of-the-art deep learning methods based on confidence maps, instead of object detection approaches, could be capable of identifying single tree-species in highly dense areas using RGB images. Methods that could accurately identify a species, among others, may help optimize several applications in environmental planning and forest management.

In the remote sensing field, the identification of palm trees with deep neural networks is a recent topic. A study performed by^52^ investigated the performance of the RetinaNet^47^ to conduct a region-wide spatial inventory of palm trees in an urban environment with high-resolution aerial RGB imagery. In this scenario, the object detection method was appropriate to singularly count palm trees. Another study conducted by^53^ evaluated the performance of another traditional deep learning method, YOLOv3^46^, to map individual palm trees using aerial imagery acquired with UAVs. As such, dealing with this type of object detection in remote sensing data has demonstrated potential, but it its lacks further investigations in highly-dense environments, such as natural forests. The traditional object detection approach may not be entirely appropriate for this environment. Because of that we propose an approach that, while being able to deal with this environment, can return improved evaluation metrics.

In the presented context, this paper presents a deep learning approach to detect individual fruit species of palm trees (*Mauritia flexuosa*; L.f. Buriti) with only aerial RGB orthoimages. As contribution of this approach, a framework to identify and geolocate single species in a high-complex forested environment is demonstrated. The study compares the performance of the proposed method with other state-of-the-art object detection deep neural networks to test its robustness. The palm tree *M. flexuosa* is a valuable source of food, remedy, fiber, and light wood for both indigenous communities and local populations^54,55^. It is also considered a native species of the Brazilian flora with both current and potential high economic values^54,56^. Besides, this species has its ecological importance, constituting a food source, nest site, and habitat to a wide variety of species and provides multiple ecosystem services^55^, which highlights the need to accurately map this species.

## Results

### Validation of the parameters

The proposed approach parameters $\sigma _{min}$, $\sigma _{max}$ , and the number of stages T, are responsible for refining the prediction map. Initially, the influence of these parameters was evaluated on the *M. flexuosa* palm trees validation set. Table 1 shows the evaluation of the number of stages T used in the MSM refinement phase. In this experiment, the values of $\sigma _{min}$ = 1, $\sigma _{max}$ = 4 and ranges T from 1 to 5 were set, and it was discovered that T = 4 achieved the best performance among the number of analyzed stages, reaching an MAE of 0.852 trees and an F1-measure of 87.1%.

**Table 1:** Influence of the number of stages (T) in counting and detection of *M. flexuosa* palms-trees ($\sigma _{min}$ = 1 and $\sigma _{max}$ = 4 were adopted).

Stages (T)	MAE	Precision (%)	Recall (%)	F1-measure (%)
1	0.933	85.1	86.4	83.8
2	0.943	93.5	83.6	86.9
4	0.852	91.5	85.5	87.1
5	0.966	93.9	83.1	86.6

The values of $\sigma _{min}$ and $\sigma _{max}$ applied in the refinement stage were also evaluated. For this, the number of stages T = 4 was adopted in the subsequent steps since it obtained the best results in the previous experiment (see Table 1). Since the $\sigma _{min}$ values represent the dispersion of the density maps around the center of the trees, it was found that smaller values do not correctly cover the trees and, therefore, can impair the detection. On the other hand, higher $\sigma _{min}$ values are also harmful as they cover more than one tree per area. Thus, the best results were obtained with $\sigma _{max} = 4$, indicating that it fits better with the *M. flexuosa* palms-trees characteristics, and generates a more accurate refinement map.

Table 2 presents the evaluation of different values of $\sigma _{min}$ responsible for the last stage of the MSM. For this, $\sigma _{max} = 4$ and T = 4 were adopted since they obtained better results in the previous experiments (Tables 2 and 3). When $\sigma _{min} = 1$, the proposed approach returned the best performance among the analyzed values. Therefore, the refinement step implemented with values of $\sigma _{min} = 1$, $\sigma _{max} = 4$, and T = 4 generated a more accurate refinement to the validation set.

**Table 2:** Influence of the $\sigma _{max}$ in counting and detection of *M. flexuosa* palms-trees ($\sigma _{min}$ = 1 and stages T = 4 were adopted).

$\sigma _{max}$	MAE	Precision (%)	Recall (%)	F1-measure (%)
3	0.931	86.7	88.7	85.8
4	0.852	91.5	85.5	87.1
5	1.611	91.4	69.6	76.8

**Table 3:** Influence of the $\sigma _{min}$ in counting and detection of *M. flexuosa* palms-trees ( $\sigma _{max}$ = 4 and stages T = 4 were used).

$\sigma _{min}$	MAE	Precision (%)	Recall (%)	F1-measure (%)
0.75	1.879	90.7	64.9	73.3
1	0.852	91.5	85.5	87.1
1.5	1.012	83.9	88.9	84.3
2	1.671	72.4	91	77.6

### Comparative results between object detection methods

The proposed method returned better performance when compared with different object detection methods like Faster R-CNN and RetinaNet. The MAE, precision, recall, and F1-measure metrics were calculated for each of them, and the results are displayed in Table 4. The proposed approach achieved high precision and good F-measure values but returned a slight-lower recall value when confronted with them. Nonetheless, it is essential to consider the tradeoff in recall difference (− 6.58% from the Faster R-CNN and − 12.35% from the RetinaNet) with the precision difference (+ 14.52 from the Faster R-CNN and + 35.49% from the RetinaNet).

Since the F1-measure uses both the precision and recall values to compute the test results, it can be assumed that the proposed approach performs better and returns a better balance between true-positive predicted and true-positive rates concerning the identification of palm trees. Nonetheless, the results are consistent with recent literature where object detection applications were applied for the identification of single tree-species^6,7,57,58^; but performed in the non-RGB image domain. The low precision values for the bounding box method may be explained by a high density of objects (i.e., *M. flexuosa* palm trees). This condition is described as problematic for deep networks based on these characteristics, especially when the boxes have high intersections with similar objects^59^.

**Table 4:** Comparative results between our method and Faster R-CNN and RetinaNet.

Method	MAE	Precision (%)	Recall (%)	F1-measure (%)
Faster R-CNN	0.984	79	90.8	81.7
RetinaNet	3.761	58	96.6	69.8
Proposed method	0.758	93.5	84.2	86.9

To verify the potential of the proposed approach in real-time processing, a comparison of its performance with other state-of-the-art methods was conducted. Table 5 shows the average processing time and standard deviation for 100 images of the test set. The values of $\sigma _{min}$ = 1, $\sigma _{max}$ = 4 and T = 4 were used to obtain the best performance in previous experiments. The results show that the approach can achieve real-time processing, delivering image detection in 0.073 seconds with a standard deviation of 0.002 using a GPU. Similarly, RetinaNet and Faster R-CNN methods obtained an average detection time and standard deviation of 0.057, 0.046, and 0.002, 0.001, respectively.

**Table 5:** Processing time evaluation of the analyzed approaches.

Method	CPU		GPU	
	Average time (s)	SD	Average time (s)	SD
Faster R-CNN	1.57	0.031	0.05	0.001
RetinaNet	1.93	0.028	0.06	0.002
Proposed Method	1.26	0.051	0.07	0.002

**Figure 1:**
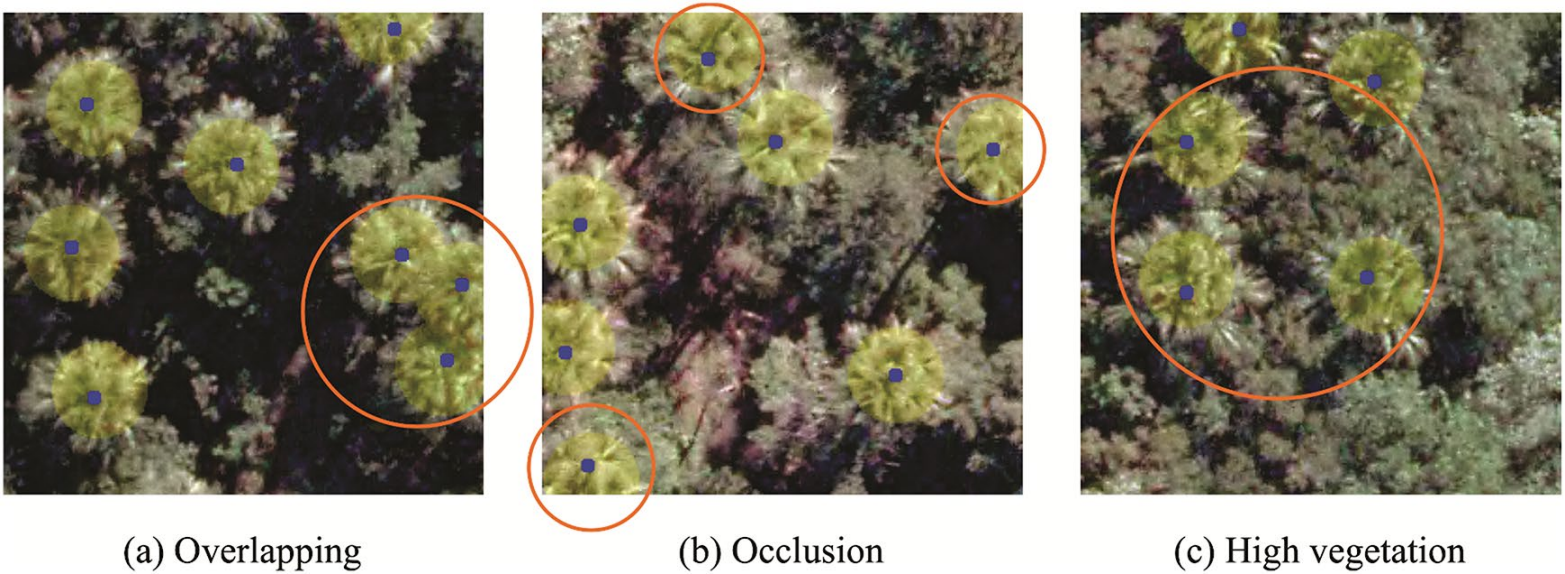
Qualitative results of the proposed method in three scenes: (**a**) an example of the detected nearby trees with overlapping, (**b**) detected trees with parts of the canopy occluded at the edge of the image, and (**c**) demons detected trees in areas of high vegetation. The orange circles highlight the detections.

Figure 1 presents the qualitative results of the proposed method where the annotations of *M. flexuosa* palm trees are marked with yellow circles, and the blue dots indicate the correctly detected positions. This approach correctly detects the *M. flexuosa* palm trees in different capture conditions, such as overlapping trees (Fig. 1a), partial occlusion of the treetops (Fig. 1b), and highly dense vegetation areas (Fig. 1c), highlighted by orange circles. Moreover, the predicted positions have a satisfactory level of accuracy, generating detection (blue dots) close to the annotations (center of the yellow circles).

**Figure 2:**
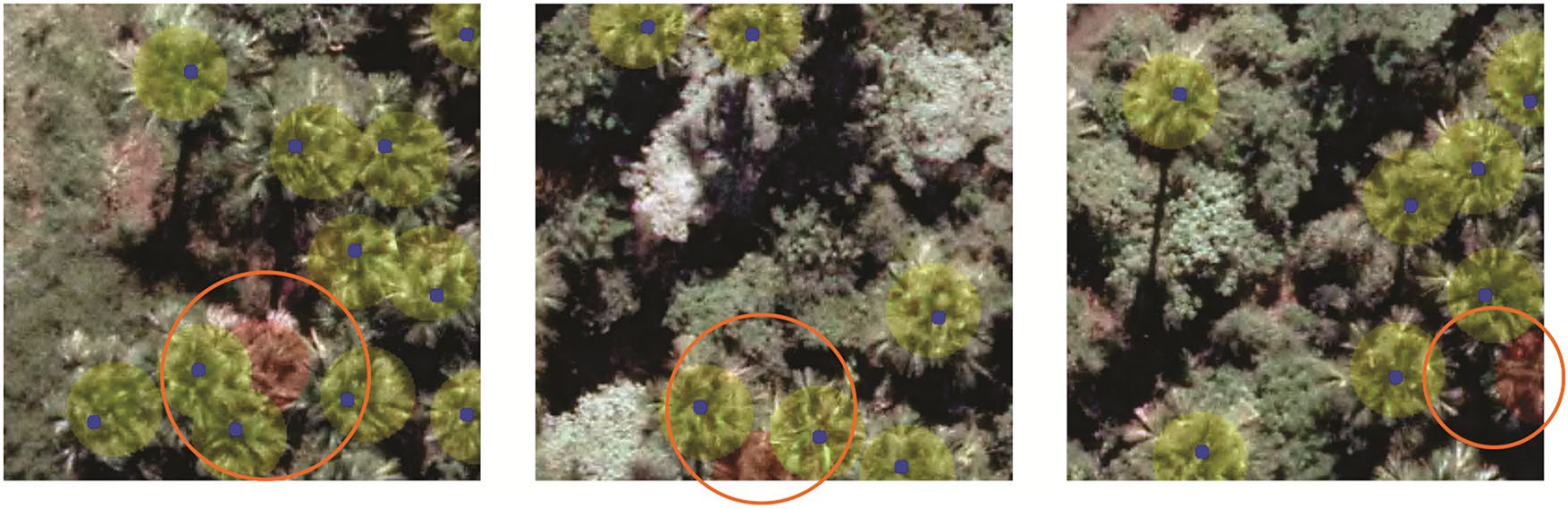
Examples of the challenges faced by our method in the *M. flexuosa* palm tree detection task. The orange circles indicate challenging detections.

Although the method obtained good results in the detection of *M. flexuosa* palm trees, it faces some challenges. Figure 2 presents areas where the incorrect detections are shown by the red circles. The main challenge is the detection of trees with a high level of occlusion at the image boundary or by overlapping of trees (highlighted by the orange circles). However, even in these few cases, the proposed approach can correctly detect most of the palm trees.

**Figure 3:**
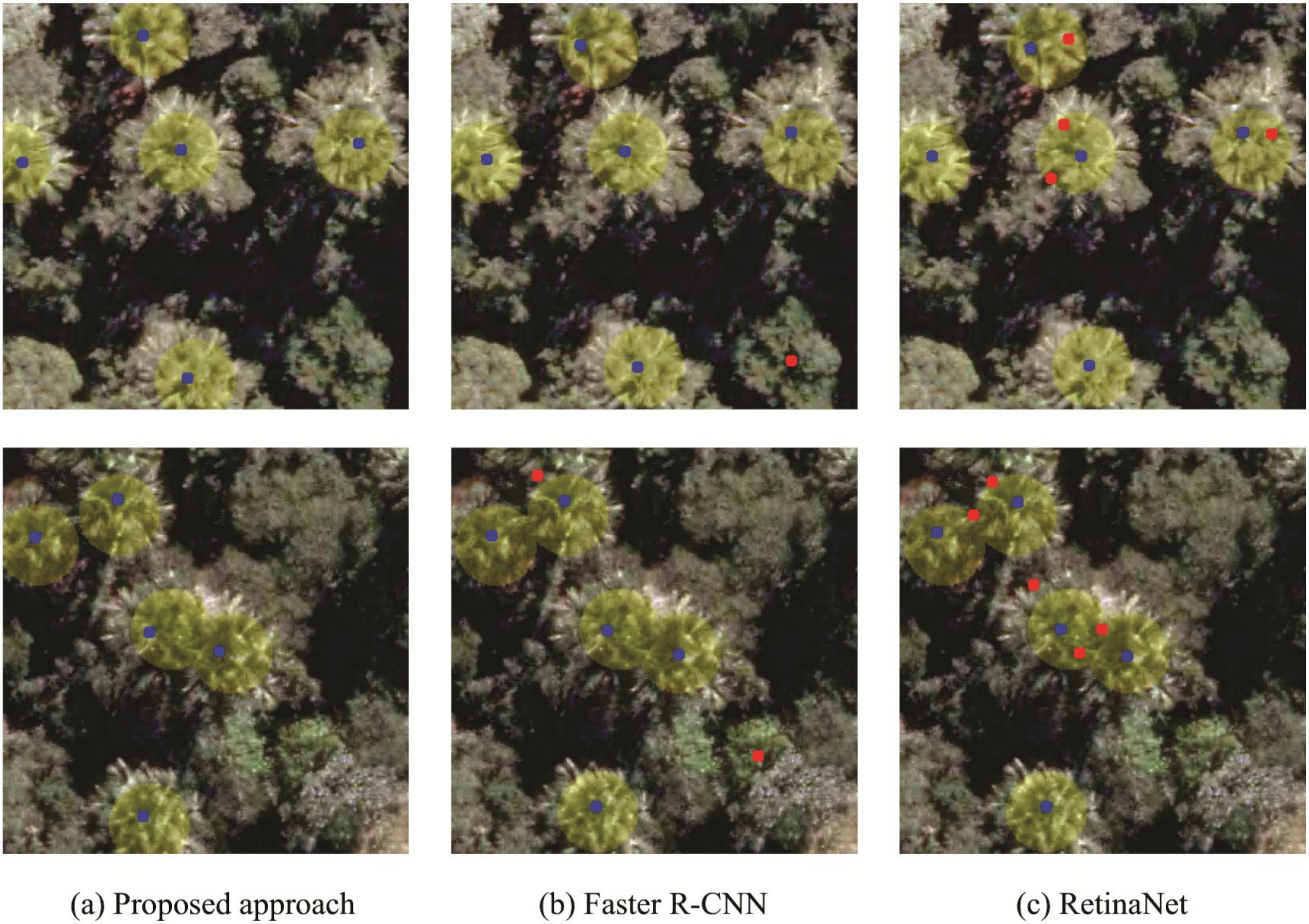
Visual comparison of the analyzed methods. (**a**) shows the detections obtained by the proposed approach; (**b**) indicates the detections of the Faster R-CNN and; (**c**) demonstrates the detections of the RetinaNet. Blue and red points correspond to correct and incorrect detection positions, respectively, and the yellow circle to *M. flexuosa* palms-trees annotation.

The visual comparison of the palm tree detection approaches is shown in Fig. 3. Column (a) displays the detections obtained by the proposed method, while Columns (b) and (c) are related to the compared methods: Faster R-CNN and RetinaNet, respectively. The approach that obtained the worst result was RetinaNet (Fig. 3c), generating many false-positives (red dots) close to the *M. flexuosa* palm trees detections. On the other hand, Faster R-CNN (Fig. 3b), despite having fewer false-positives, did not properly learn the characteristics of the palm trees and incorrectly detected other tree species among them. Following the quantitative results shown in Table 4, the proposed approach has the greater precision in detecting *M. flexuosa* palm trees (Fig. 3a), while having the least number of incorrect detections (false-positives).

## Discussion

This study demonstrated a feasible method to automatically map single palm tree species of the *M. flexuosa* plant genus using an RGB imagery dataset. *Mauritia flexuosa* frequently occurs at low elevations, with high density on river banks and lake margins, around water sources, and in inundated or humid areas^56^. This is one of the most widely distributed palm trees in South America, Brazil. This species occurs in the Amazon region, Caatinga, Cerrado, and Pantanal, and is one of the palm trees mostly used by humans, being an important item in the diet of many indigenous groups and rural communities^56^.

Mapping *M. flexuosa* palm trees is an important practice for multiple regions of South America, like Brazil, where this plant is viewed as a valuable resource. This palm is widely used for several purposes, is considered a species of multiple use^54^, occurs in areas of “Veredas”, considered protected by the Brazilian forest code, but there is still a great lack of characterization of the habitats of this species in this country. Mapping and identifying populations of palm *M. flexuosa* is relevant because it is a reliable indicator of water resources, such as streams inside dense gallery forests, slow-flowing swamp surface water, and shallow groundwater in the Cerrado region, vital for humans and wildlife, besides being a valuable source of several non-timber forest products. The approach provides useful information for sustainable economic use and conservation.

As described, single tree species identification is a challenging task even for state-of-the-art deep neural networks when considering only RGB imagery. Mainly because forest environments are constituted by multiple spectral spatial information, overlapping canopies, leaves and branches, size, growth stages, and density, among others. In this manner, studies considered different data to help solve this issue like density point information, canopy height, digital terrain and surface models, spectral divergence, etc.^4,25,34,45^. Regardless, in this paper, it is proposed a simplification of this process by adopting little input information (i.e., label features such as points and RGB imagery) and a robust method that once trained, can rapidly perform and resolve the said task even in a real-time context.

The results of the present approach achieved satisfactory precision (93.5%), recall (84.2%), and F1-measure (86.9%) values, respectively), and a small MAE (0.758 tree). Studies that applied deep neural networks for detecting other types of arboreal vegetation returned approximated metrics. For the identification of citrus-tree, a CNN method was able to provide 96.2% accuracy^13^, and in oil palm tree detection, a deep neural network implementation returned an accuracy of 96.0% (Li et al., 2019). One different kind of palm trees than the ones evaluated in our dataset was investigated with a modification of the AlexNet CNN architecture and returned high prediction values (R = 0.99, with the relative error between 2.6 and 9.2%)^57^. A study^7^ achieved an accuracy higher than 90% to detect single tree-species using the RetinaNet and RGB images. However, in the aforementioned papers, the tree density patterns are differently from ours, and the evaluated individual trees are more spaced from each other, which makes a simpler object detection problem.

In the described manner, the proposed method may help in mapping the *M. flexuosa* palm tree with little computational load and high accuracy. Since this approach can compute point features as labeled objects, it reduces the amount of label-work required from the human counterpart. Additionally, the method provided a fast solution to detect the palm tree’s location with a delivering image detection of 0.073 seconds and a standard deviation of 0.002 using a GPU. This information is essential for properly calculating the cost and effectiveness of the method. The presented approach may help new research while providing primary information for exploring environmental management practices in the experiment context (i.e., evaluating a keystone tree species). The proposed method could also be incorporated into areas and regions to help detect the *M. flexuosa* palm tree and contribute to decision-making conservation measures of the said species.

## Conclusion

This paper presents an approach based on deep networks to map single species of fruit palm trees (*Mauritia flexuosa*) in aerial RGB imagery. According to the performance assessment, the method returned an MAE of 0.75 trees and F1-measure of 86.9%. A comparative study also shows that the proposed method returned better accuracy than state-of-the-art methods like Faster R-CNN and RetinaNet under the same experimental conditions. Besides, this approach took a shorter time to detect the palm trees with 0.073 seconds for delivering image detection and achieved a standard deviation of 0.002 using the GPU. In future implementations, it should be possible to add new strategies in this CNN architecture to overcome challenges regarding other tree patterns. Still, the identification of individual species can help to assist in both monitoring and mapping important singular species. As such, the proposed method may assist in new research for the forest remote sensing community that includes data obtained with RGB sensors. As a future study, different takes on the detection approach could be implemented to enhance the precision of the method, one of which being the investigation of different loss functions and approaches to detect each tree.

## Methods

**Figure 4:**
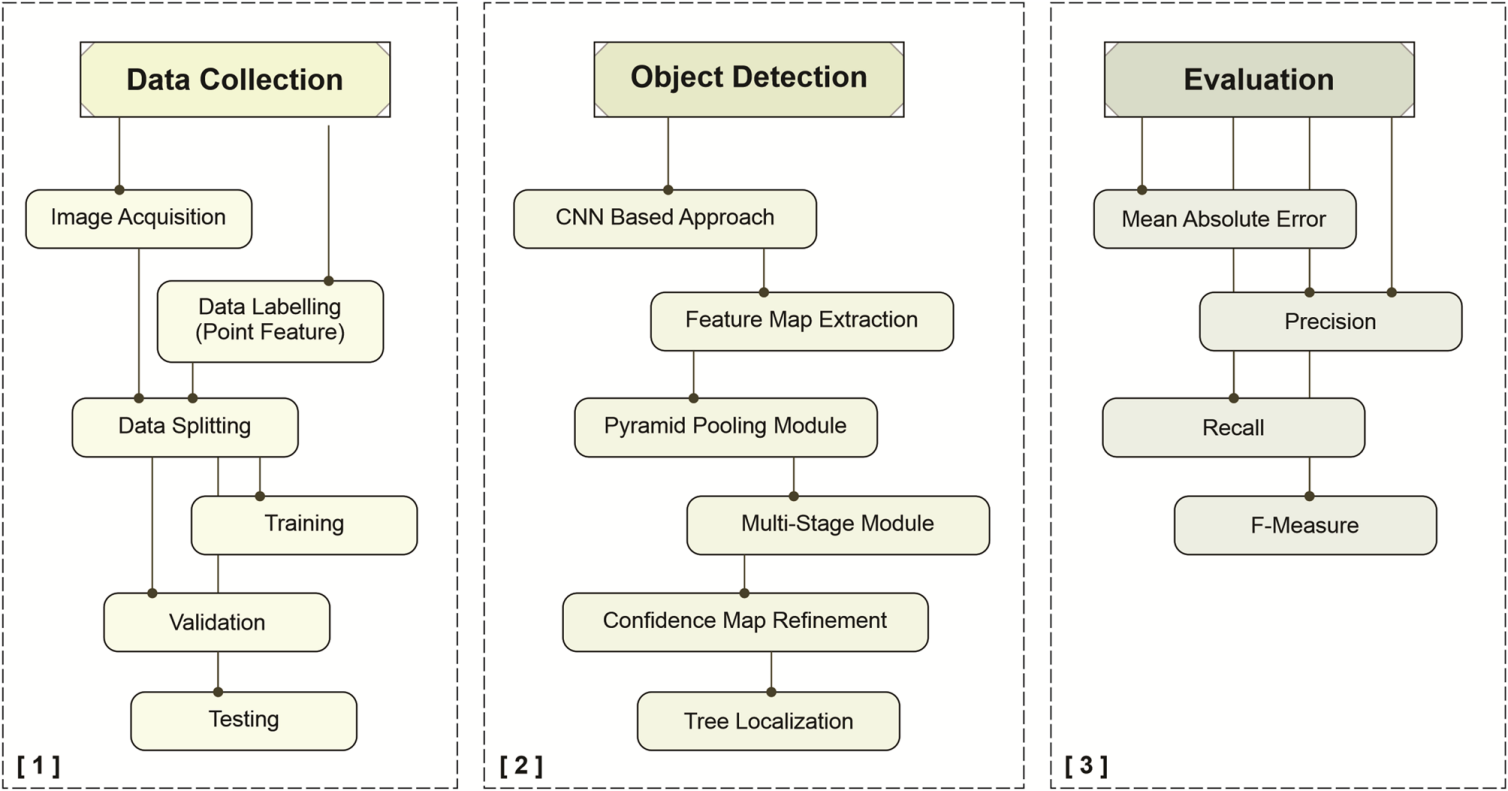
Summarized phases of the proposed approach.

The method proposed in this paper is composed of three main phases (see Fig. 4): (1) the dataset was composed of aerial RGB orthoimages obtained from a riparian zone of a well-known populated region of *M. flexuosa* palm trees. With specialist assistance, the palm trees in the RGB images were visually identified and labeled in a Geographical Information System (GIS). The image and labeled data were split into groups of training, validation, and testing subsets; (2) the object detection approach was applied in a computational environment; (3) the performance of the proposed method was compared with other networks.

### Study area and mapped species

**Figure 5:**
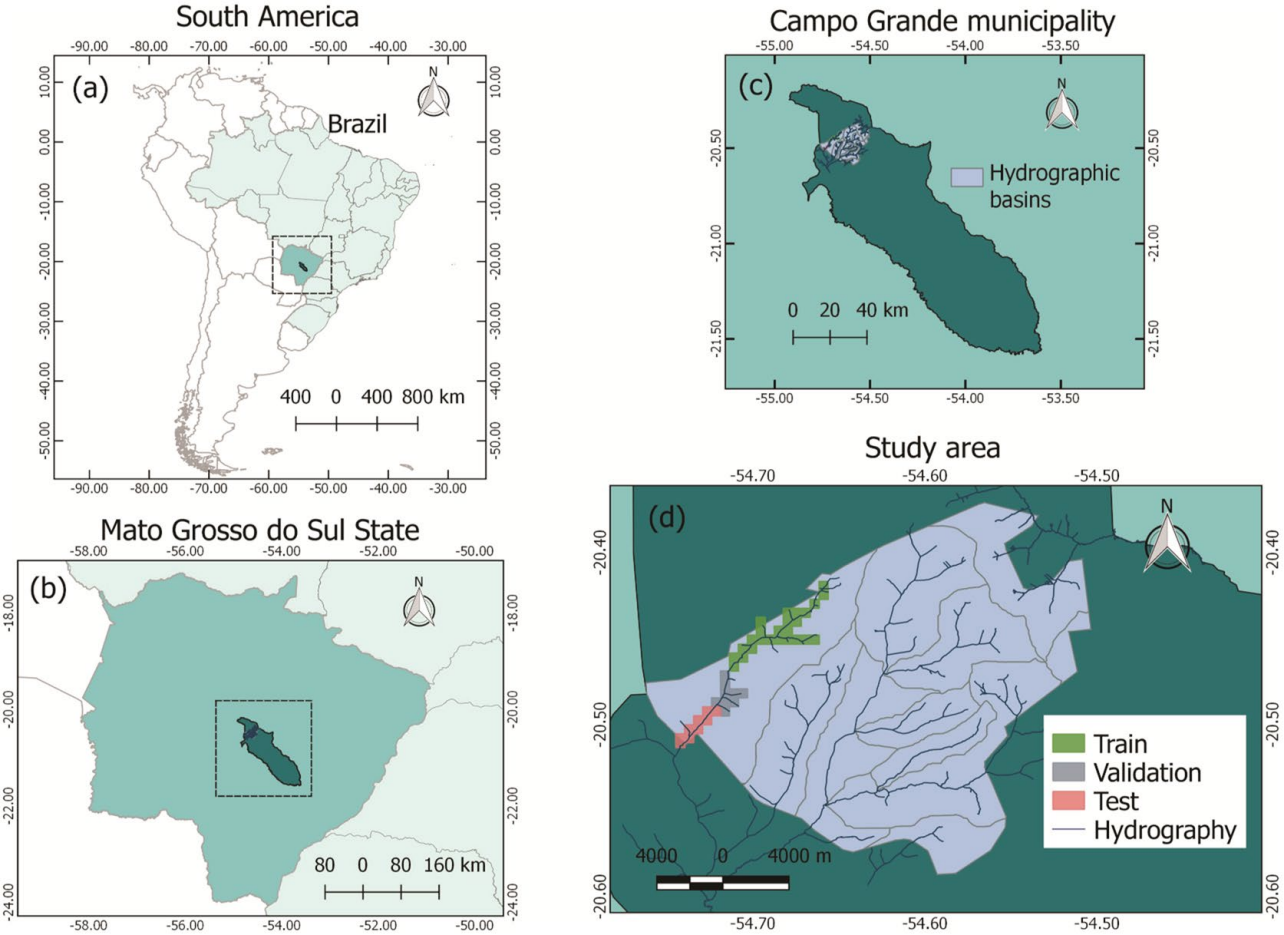
Location map of the study area in (**a**) South America and Brazil, (**b**) Mato Grosso do Sul, (**c**) Campo Grande, and (**d**) study area. Map created with QGIS 3.16.9: https://qgis.org/en/site/.

The riparian zone of the upper-stream of the Imbiruçu brook, located near the city of Campo Grande, in the state of Mato Grosso do Sul, Brazil was selected for the study (Fig. 5). This stream, formed by a dendritic drainage system, is inserted in the hydrographical basin of the Paraguay River and covered by the Cerrado (Brazilian Savanna) biome. This area is composed of a highly complex forest patch containing a widespread of palm tree species *Mauritia flexuosa* (popular name Buriti). The Arecaceae is a dioecious^60^ long-living species and grows naturally in flooded areas, providing water balance for rivers and other water bodies. In highly dense, monodominant stands in flooded areas, mature *M. flexuosa* palm trees reach 20 m high^60^. The evaluated site in our experiment, in specific, is one of the well-known locations where a large number of samples of this species is sufficient to train a deep neural network.

**Figure 6:**
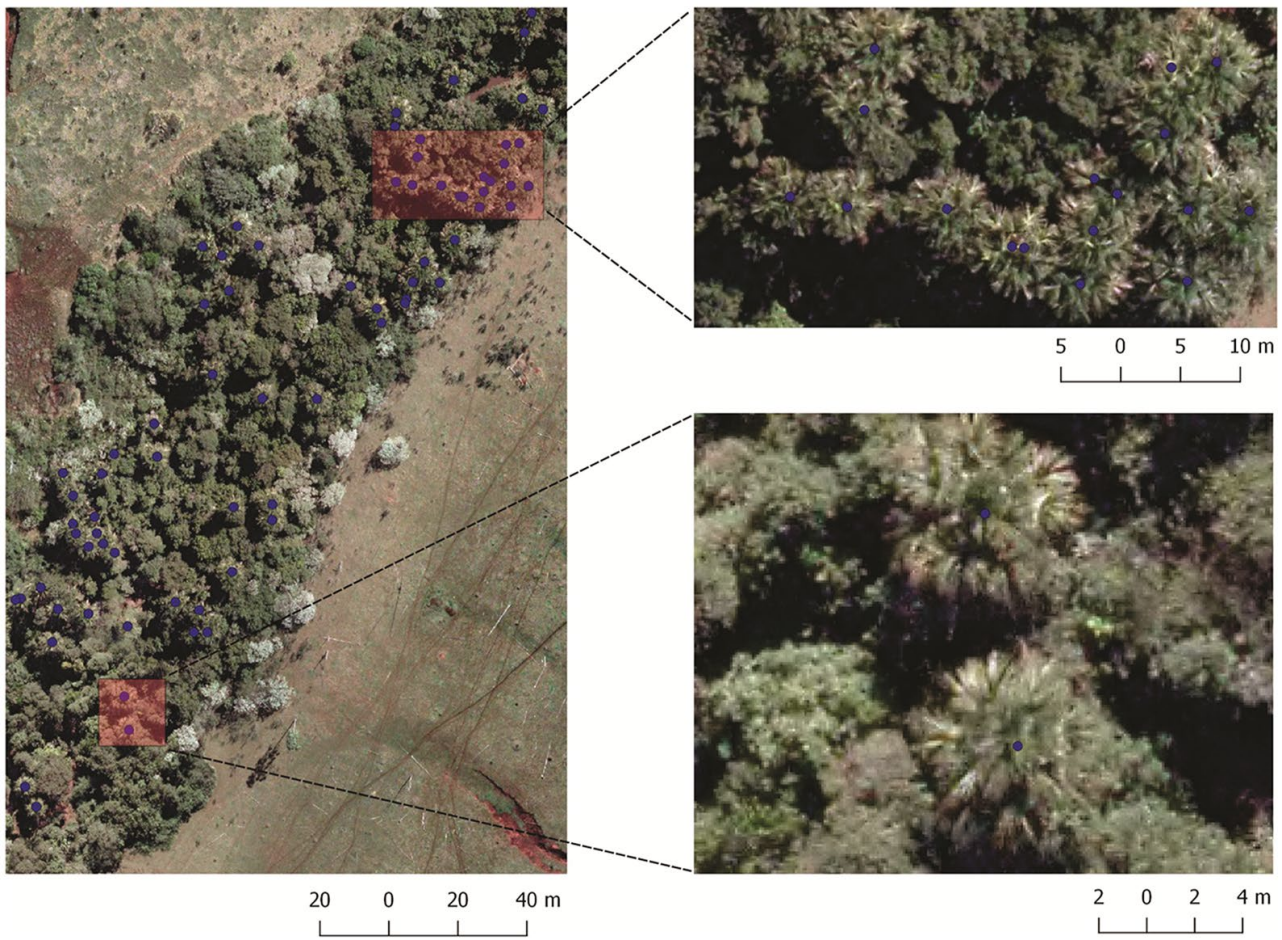
Examples of the labeled dataset. *M. flexuosa* palm trees are represented with blue dots.

The aerial RGB orthoimages were provided by the city hall of Campo Grande, State of Mato Grosso do Sul, Brazil. The ground sample distance (GSD) of the orthoimages is 10 cm. A total of 43 orthoimages with dimensions 5619 × 5946 pixels were used in the study. This aerial image dataset was composed of 1394 scenes, where 5334 palm trees were manually labeled and used as ground-truth (Fig. 6).

### Proposed method

**Figure 7:**
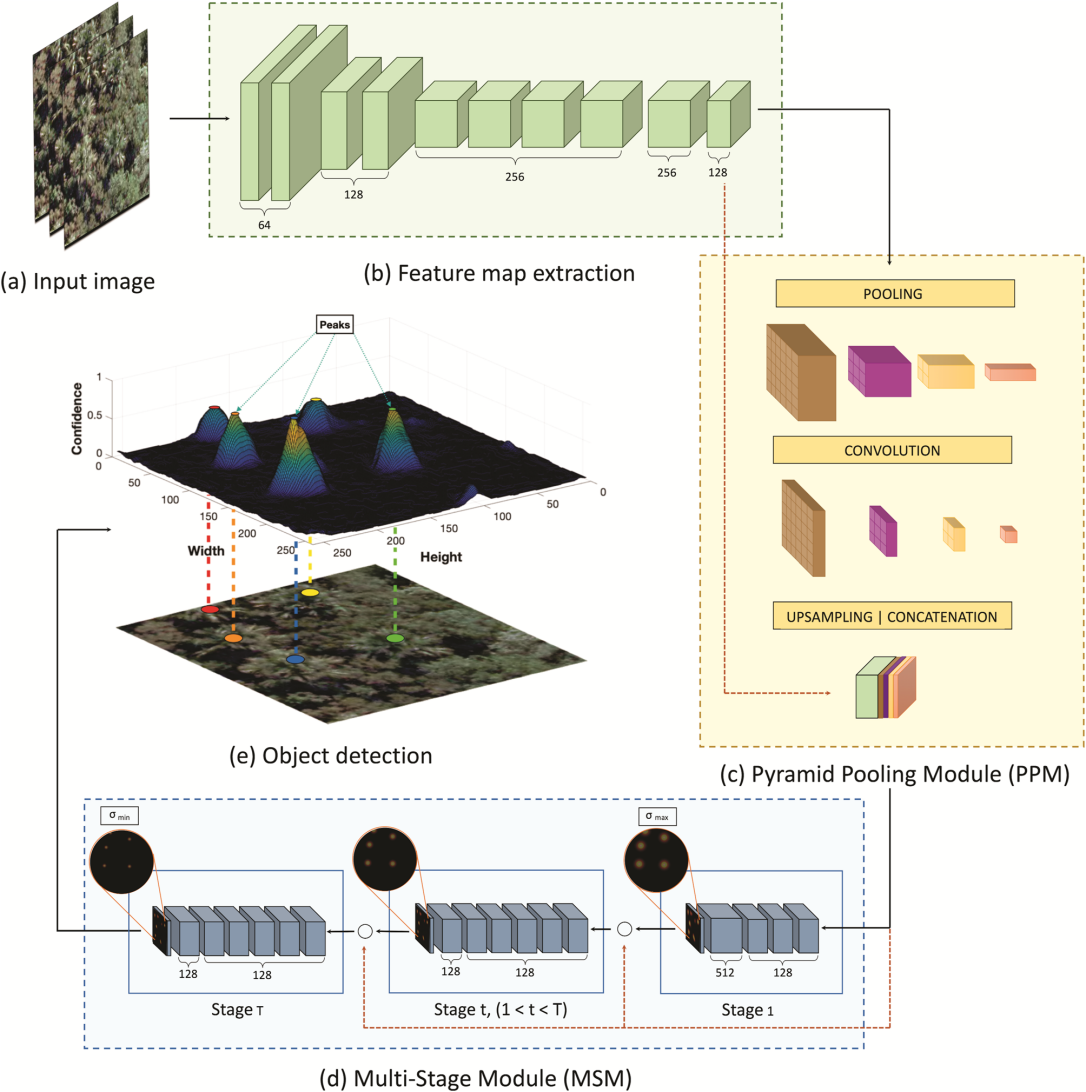
Proposed CNN. The feature map (**b**) is extracted from the input image (**a**) and improved by the PPM (**c**). The result is used as input at the MSM step (**d**), where T stages enhance the prediction positions (**e**).

This study proposes a CNN method that uses the RGB image as an input and, throughout a confidence map refinement, returns a prediction map with tree locations (Fig. 7). The objects’ position is calculated after a 2D confidence map estimation, based on previous works^58^. The first step of the architecture extracts the feature map (Fig. 7a). In a sequential step, the feature map goes through the Pyramid Pooling Module (PPM)^61^. The last step of the architecture produces a confidence map in a Multi-Stage Module (MSM)^58^ that enhances the position of the tree by adjusting the prediction to its center.

### Feature map extraction and PPM

For the feature map extraction (Fig. 7b), the proposed CNN is based on the VGG-19^49^. Here, the network is composed of 8 convolutional layers with 64, 128, and 256 filters with a 3 × 3 size window, with Rectified Linear Units (ReLU) functions in all layers. The spatial volume size was reduced in half after the second and fourth layers by a max-pooling layer (2 × 2 window). The PPM^61^ was used (Fig. 7c) to extract global and local information, which helps the CNN to be less variant to tree scale differences. The extracted features are upsampled to size equivalent to the input feature map and concatenated with it to create an enhanced version of the image.

### Tree localization

The MSM step (Fig. 7d) estimates the confidence map from the feature map extracted in the previous module. The MSM is composed of T refinement stages, where the first stage contains 3 layers of 128 filters with 3 × 3 size, 1 layer with 512 filters of 1 1 size, and one final layer with 1 filter that generates the confidence map C1 from the first stage. The position of the trees predicted in the first stage is refined in the T − 1 stages. In each stage t $\in$ [2, 3,…, T], the prediction (C) is returned from a previous stage (t − 1) and the feature map from the PPM module is concatenated. The final layer in this step has a sigmoid activation function since the method considers the probability of occurrence of a tree to exist or not [0,1]. The concatenation process allows for both global and local context information to be incorporated in it. At the end of each stage, a loss function (1) is adopted to avoid the vanishing gradient problem. The general loss function is calculated by the following Eq. (2).

1 $$\begin{aligned} f_t=\sum _{p}{{\parallel {\hat{C}}}_t\left( p\right) -C_t\left( p\right) \parallel _{2^\prime }^2} \end{aligned}$$

where ${\hat{C}}_t$(p) is the ground-truth confidence map of location (p) in stage (t).

2 $$\begin{aligned} f=\sum _{t=1}^{T}f_t \end{aligned}$$

**Figure 8:**
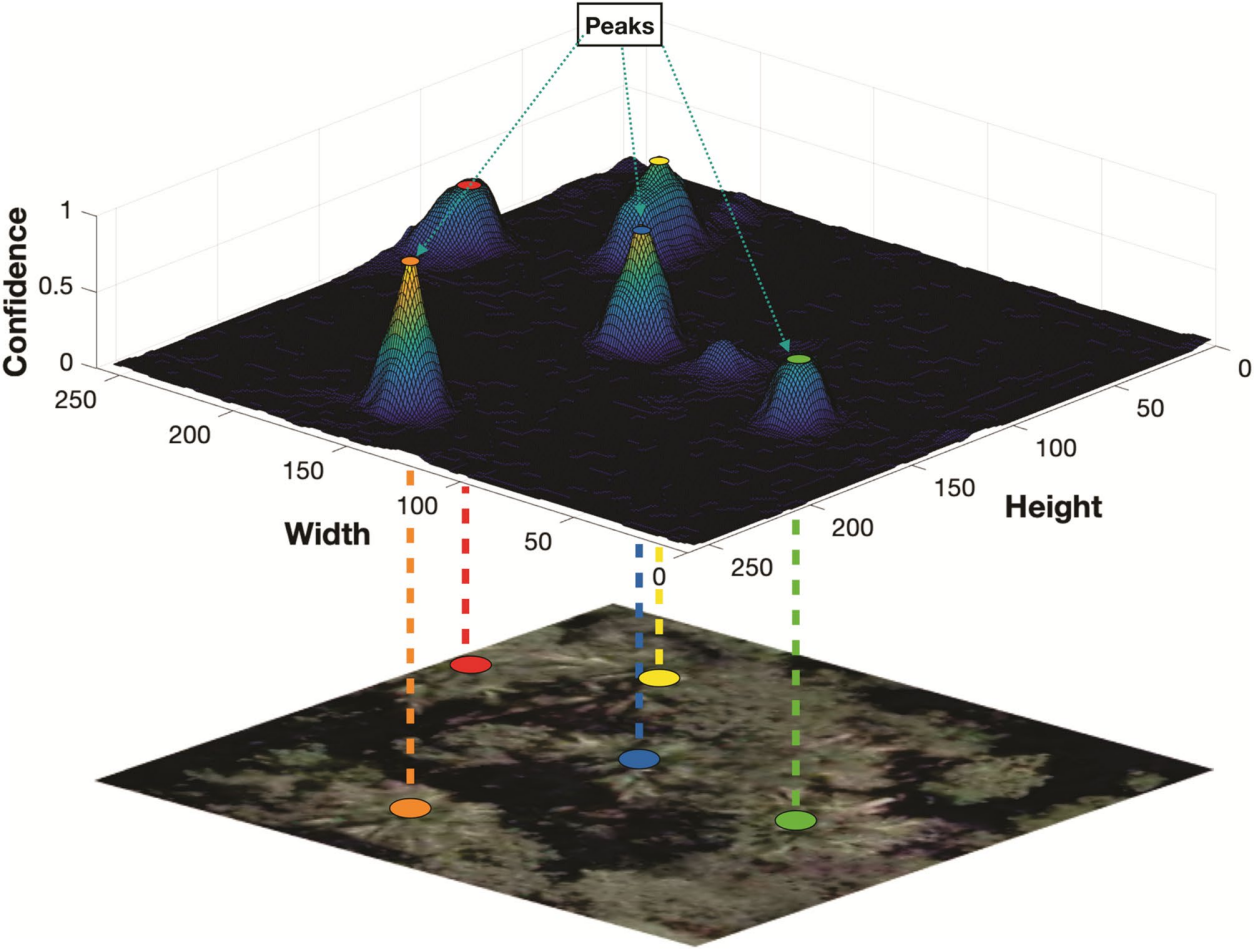
Tree localization example from a refined confidence map. Figure was created with Matplotlib 3.4.2: https://matplotlib.org/.

The confidence map is generated by a 2D Gaussian kernel at the center of the labeled tree. A standard deviation $\sigma _t$ controls the spread of the peak for each Gaussian kernel (Fig. 8). Different values of $\sigma _t$ were used to refine the predictions. The value of $\sigma _1$ in the MSM is set to maximum ($\sigma _{max}$) while the $\sigma _t$ in the final stage is set to minimum ($\sigma _{min}$). In the early phases of the experiment, different values for t were adopted to improve its robustness. Finally, the tree location is estimated by the peaks of the confidence map (Fig. 8). These peaks are considered the local maximum of the confidence map, representing a high probability of a tree occurrence. P = ($x_p$, $y_p$) is considered as a local maximum if $C_T (p) > C_T (v)$ for all neighbors v. Here, v is given by ($x_p \pm 1$, $y_p$) or ($x_p$, $y_p \pm 1$).

A peak in the confidence map is defined as a real tree if $C_T$(p) $> \tau$ (Fig. 7e). To prevent the network from confusing trees in a nearby range from each other, a distance of $\delta$ is defined. For this study, $\tau$ equal to 1 pixel and $\delta$ equal to 0.35 were defined as valid metrics. These values were defined during a previous experimental phase.

**Figure 9:**
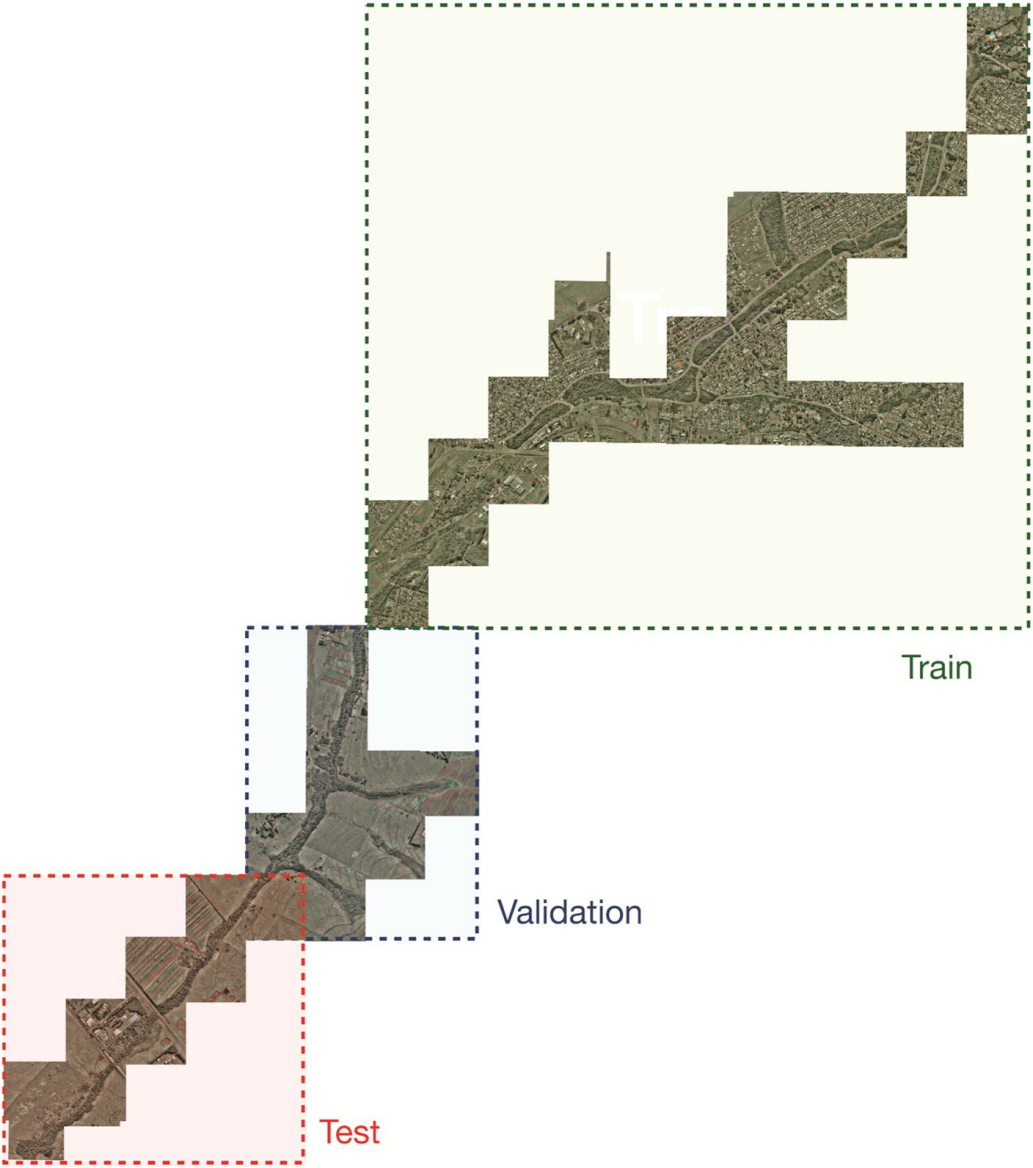
Training, validation, and testing datasets separated per region. Figure created with QGIS 3.16.9: https://qgis.org/en/site/.

### Experimental setup

For the experimental setup, the RGB ortohomosaics were separated into training, validation, and testing, respectively (Fig. 9). They were split into nonoverlapping patches of 256 × 256 pixels (25.6 m × 25.6 m). The patches were then divided into training (42.3%), validation (34.5%), and testing (23.2%) sets. Table 6 lists the number of samples (trees) and image patches, and Fig. 9 displays examples of the orthomosaics used to extract the datasets. For the training process, the CNN was initialized with pre-trained weights from ImageNet and a Stochastic Gradient Descent optimizer was applied with a moment equal to 0.9. For this, the validation set was used to adjust the learning rate and the number of epochs, which were set to 0.001 and 100, respectively.

**Table 6:** Description of the training, validation, and testing sets of the *M. flexuosa* palms-trees dataset.

Dataset	Number of patches	Number of samples (palm trees)
Training	590	1784
Validation	481	2296
Testing	323	1254

The performance of the proposed network was assessed with the following metrics: mean absolute error (MAE); precision (P); recall (R), and; F1-measure (F1). The results were compared with Faster R-CNN and RetinaNet methods. Since these methods are based on bounding boxes, the plant position (x, y) from the labeled ground truth was used as a center of the box. The correct size of the box corresponds with the size occupied by the tree canopy. To perform this comparison, the same conjuncts of training, validation, and testing datasets were adopted for the three methods. Likely, an inverse process was applied during the test phase, where the position of the tree was obtained by the center of the point inside the predicted bounding-box of the RetinaNet and Faster R-CNN methods. This allowed applying the same metrics (MAE, P, R, and F1) for comparing the performances of each neural network.
